# Discrepancies between Genetic and Visual Coat Color Assignment in Sarcidano Horse

**DOI:** 10.3390/ani14040543

**Published:** 2024-02-06

**Authors:** Maria Consuelo Mura, Vincenzo Carcangiu, Giovanni Cosso, Nicolò Columbano, Eraldo Sanna Passino, Sebastiano Luridiana

**Affiliations:** 1 Department of Veterinary Medicine, University of Sassari, Via Vienna 2, 07100 Sassari, Italy; vcarcangiu@uniss.it (V.C.); ncolumbano@uniss.it (N.C.); esp@uniss.it (E.S.P.); sluridiana@uniss.it (S.L.); 2 Agris Sardegna, 07100 Sassari, Italy; gcosso@agrisricerca.it

**Keywords:** basic coat color definition, offspring’s coat color prediction, phenotypic distribution, Sarcidano Horse pigmentation

## Abstract

**Simple Summary:**

Since horses were domesticated, human selection has introduced new coat colors and patterns, which have become a feature of added value in many current breeds. The wild population generally exhibit a basic coat color set (Bay, Black and Chestnut) due to a lack of crossbreeding with domestic breeds and mating with subjects carrying the same color set. Sometimes, visual identification of the coat color is difficult due to several individual and environmental conditions, leading to incorrect registration. However, it is crucial to identify coat colors accurately and to correctly report them in the Stud Book for legal and medical certification. Furthermore, Grays are horses born with an original basic coat color, but, gradually, hair graying leads to the complete loss of coat pigmentation with age. Therefore, it becomes very difficult to predict what color foals will be born from a Gray parent. Indeed, many other traits, like behavior, athletic skills, and genetic diseases, could be influenced by coat color. For breeders who want to produce foals with specific coats, this feature is also central. For all these reasons, molecular analysis of two major genes involved in the basic coat color definition (*MC1R* and *ASIP* genes) was conducted on 90 Sarcidano Horses, to correctly identify and assign individual coat colors.

**Abstract:**

This study aimed to evaluate the discrepancies between genetic and visual coat color assignment in the Sarcidano Horse and to elucidate potential reasons. Individual DNA from 90 Sarcidano Horses was used for genetic assignment of coat color to explore the correspondence with individual forms containing phenotypical traits. The *MC1R* exon 1 and *ASIP* exon 3 have been genotyped and sequenced to obtain a picture of the coat color distribution in this breed. Surprisingly, once we compared the genetic results with the individual forms reporting the phenotypic data for each subject, a certain degree of non-correspondence between the phenotypic and genetic data in relation to coat color emerged. From the genetic analysis, Chestnuts (*n* = 58) resulted the most common Sarcidano Horse (*n* = 58), followed by a quite large number of Blacks (*n* = 28) and a very small number of Bays (*n* = 4), whereas phenotypic distribution resulted in 38 Chestnuts, 40 Bays, only 2 Blacks, and 10 Grays (without the possibility of recognizing the true color they carried). Chestnut resulted a very representative coat color, while many horses that visually identified as Bays were genetically Blacks. This discrepancy, that could be due to a variety of individual and external factors, including age, time of year, living situation and dietary condition, suggesting the importance of accurate coat color identification to ensure adequate features registration and reliable prediction of offspring’s coat color.

## 1. Introduction

The Sarcidano Horse is an autochthonous, semi-feral breed of small horses native to Sarcidano, a region in the heart of Sardinia (Italy). It belongs to the 26 minor Italian breeds of limited diffusion collected in the Stud Book, managed, since 2018, by ANAREAI, the national association for local horse and donkey breeds [1]. These horses are locally known for their sturdy build and were traditionally used for rural work and riding. They live in conditions of geographical isolation [2,3,4], due to insularity and to the wild living environment, and are recognized for their endurance and adaptability to the local terrain. According to the breed standard, Sarcidano Horses typically come only in basic coat colors, Chestnut, Bay, Black and sometimes Gray, without spotting or color dilution. Gray horses have a basic coat color at birth, but as they age, the graying of their hair eventually causes the entire coat to lose its pigmentation [5,6,7]. The trait of graying with age is inherited in an autosomal dominant pattern, so it can be difficult to visually identify the real color of a Gray horse. Only two loci are involved in Chestnut, Bay and Black coat coloration: Extension locus, coding melanocortin-1-receptor (*MC1R*) gene, and Agouti locus, coding the agouti-signaling protein (*ASIP*) gene, which is an antagonist of the *MC1R* gene. The correct functioning of the *MC1R* gene produces the MC1R protein, driving eumelanin (black pigment) synthesis by melanocytes, producing Black coats [8,9,10]. Contrarily, alteration in the *MC1R* gene Exon 1, produced by a transition from C to T, leads to phaeomelanin (reddish pigment) synthesis, producing Chestnut coats [11,12]. The *ASIP* gene produces the ASIP protein, which has the power to antagonize *MC1R* gene functioning in the melanocytes located in the body but not in the extremities, thus producing Bay coats, resulting in a reddish-brown body color with black point coloration on the mane, tail, ear edges and lower legs [13]. Antagonism of *MC1R* by the *ASIP* gene is efficient only in dominant/non-mutant variants of the *MC1R* gene, whereas when the receptor is already defective, because of the C > T mutation explained above, the antagonism cannot occur, so a Chestnut coat is still produced [8].

In a previous study on this breed, we focused on the genetic distribution of coat color as a first step aimed at contributing to the general knowledge of the Sarcidano Horse from a perspective of protection of this local genetic resource [14]. The study highlighted the distribution of the genetic coat colors, pointing out that the absence of spotting or color dilution could be suggestive of low phenotypic variability and the absence of crossbreeding with other domestic breeds. Just thanks to that investigation, carried out on 70 horses, we became aware of a certain inconsistency in the color assigned by visual evaluation compared to that detected by genetic investigation. By analyzing this phenomenon in more detail and expanding the number of samples, these discrepancies reached a substantial number, suggesting that the phenotype of these horses is not always adequately recorded, thus raising awareness that this local resource should be better safeguarded to prevent its decline and disappearance. Since the genetic color is the only one that can be passed on to offspring, correct assignment of the parental coat color is essential to ensure both correct individual identification and the prediction of foal color, as stated in other studies conducted on other local breeds [15,16,17]. In a wild population, coat color provides camouflage and protection in natural environments, helping horses to blend into their surroundings and evade predators. Domestication and human selection introduced a variety of coat colors and patterns that are currently typical of specific breeds [18,19]. Diluted or spotted coats, for example, allowed easier recognition of domestic from wild animals, and were, therefore, probably sought in the early stages of domestication [20]. Thus, the study of coat color distribution could also be suggestive of possible crossing with domestic breeds, thus contributing to the observation of genetic isolation of local breeds [21]. Correct coat color registration in individual documents avoids recognition errors and allows for appropriate archiving of data, therefore granting the correct assignment of relationships to recognize family units and to predict offspring’s coat color. Although some color assignments can be correctly determined based on phenotype alone, genetic testing may be necessary to define phenotypes that are visually ambiguous and can help to determine color possibilities for foals.

Therefore, the aims of the present research were to evaluate the discrepancies between genetic and phenotypic coat color assignment in the Sarcidano Horse and to elucidate potential reasons, in order to finally propose an effective and low-cost system for correctly recording individual phenotypes.

## 2. Materials and Methods

### 2.1. Animals and Territory

**Figure 1:**
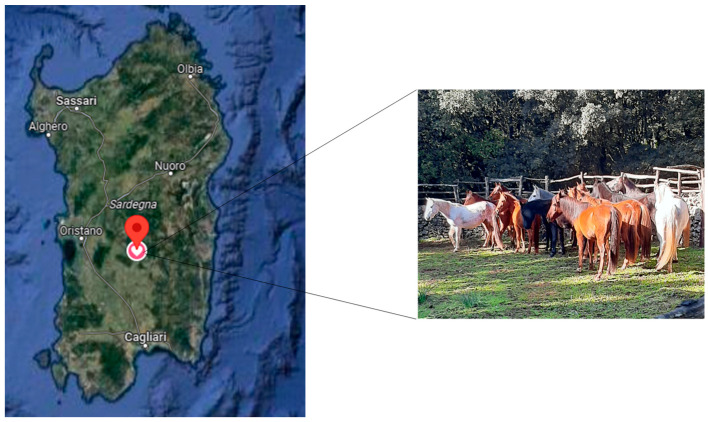
On the (**left**), image of Sardinia (Google, Immagini ©2023 TerraMetrica. Dati cartografici ©2023 Google. Inst. Geogr. Nacional) with a mark showing the municipality of Laconi where the Sarcidano Horse lives. On the (**right**), photo of a group of Sarcidano Horses (taken in December 2023) with different coats present in the population (from personal archive).

Sarcidano is the name of a plateau and district in Central-Southern Sardinia in the municipality of Laconi. The climate is influenced by the surrounding mountains, contributing to regional variation in temperature and precipitations. Summers are hot and dry, with temperatures often exceeding 30 °C, while winters are mild and relatively wet. The area has a typical Mediterranean vegetation pattern, with shrubs, olive trees and cork oaks. In this harsh environment, Sarcidano horses roam freely, grazing on natural vegetation, and are perfectly suited to the local surroundings (Figure 1).

The present study involved 90 of the approximately 120 Sarcidano Horses recorded in 2018, corresponding to 75% of the entire population registered in that year. The current number of Sarcidano Horses in the ANAREAI Stud Book is 109 heads, but the exact number could be quite different since some subjects, although microchipped, are not yet registered. This study used blood samples provided by the veterinarians who captured the Sarcidano Horses at different times, from February 2016 to January 2018 for periodic checks or extraordinary events, such as microchip implantation or the need to move animals to safer areas based on environmental conditions (frequent bushfires in summer). These horses were anesthetized intramuscularly by a single-dart injection using a combination of tiletamine, zolazepam, detomidina, and acepromazine under the supervision of a veterinary anaesthetist. While they were under anaesthesia, they were also subjected to a general objective examination (to ascertain their health conditions, the presence of wounds and parasitic infestations) and subjected to sampling of biological material (blood, hair, faeces).

More recently, since 2021, captures and sanitary checks have occurred once a year, in late fall (November–December) to individually identify and register newborns in the ANAREAI Stud Book, and to verify the sanitary conditions of the adults. The animals are grouped in small catch paddocks and taken one by one. Each horse is equipped with an electronic transponder on the left portion of the neck, which is verified with a chip reader. The individual microchip number corresponds to the phenotypic card containing the horse’s name, date of birth, sex, biometric data, and coat color. After registration, an individual identity document (green passport) is issued.

### 2.2. Amplification and Mutations Dedection

DNA was extracted from the individual whole blood samples, taken from the 90 Sarcidano Horses above, using the NucleoSpin^®^ Blood kit (Macherey-Nagel, Düren, Germany), according to the manufacturer’s protocol. The polymerase chain reaction (PCR) technique was carried out to amplify the *MC1R* gene Exon 1 and *ASIP* gene Exon 3, which are known to control base coat color set in horses. A first primer set was used to amplify a 320 base pair (bp) fragment, corresponding to part of the unique Exon 1 of the *MC1R* gene and one other primers’ pair was designed to delimit a 102/91-bp polymorphic fragment, corresponding to the entire Exon 3 of the *ASIP* gene. The primers used were those reported by Cosso et al. [6]. Both pairs of primers and all the obtained results refer to the latest horse genome version EquCab3.0 (GCF_002863925.1). A PCR reaction for both fragments was carried out in a 25 µL final volume, containing 150 ng of individual genomic DNA, 1X PCR Buffer (without MgCl_2_); 2 mM MgCl_2_; 0.2 mM dNTPs; 0.2μM primers (Bio-Fab Research srl, Roma, Italy); and 0.25 Units (U) Taq DNA polymerase (HOT FIREPol^®^ Polymerase, Solis BioDyne, Tartu, Estonia) and ultrapure water DNase/RNase free (Water PCR grade, Solis BioDyne, Tartu, Estonia) up to 25μL. Amplification conditions were as follows: 95 °C (5 min); 35 (*MC1R* gene) or 40 (ASIP gene) cycles of 95 °C (30 s), 58 °C (*MC1R*) or 60 °C (*ASIP*) (30 s), 72 °C (30 s); 72 °C (10 min). The PCR reaction was performed using MAXYGENE II thermocycler (Axygen^®^ Tewksbury, MA, USA). Amplification results were visualized after electrophoretic run (110 V for 30 min) in a 2% ultrapure agarose gel (*w*/*v*) (iNtrRon Biotechnology, Sangdaewon-Dong, Republic of Korea) added with 9μL of RedSafe stain (iNtrRon Biotechnology, Sangdaewon-Dong, Republic of Korea), in 1X TAE electrophoresis buffer, and observed under ultraviolet light (UVItec, Cambridge, UK), together with a 100-bp Ladder (GeneRuler, Thermo Scientific™, Waltham, MA, USA).

*MC1R* gene polymorphisms were detected by the restriction fragment length polymorphism (RFLP) technique, using *TaqI* endonuclease for the digestion of the obtained PCR products, thus allowing us to recognize a C with a T transition within the amplicons’ nucleotide sequence.

Instead, the polymorphism within the *ASIP* gene Exon 3 was produced by an 11 bp deletion, thanks to which it was possible to attribute the individual genotype directly through electrophoretic reading of the amplicons, producing 102 or 91 bp fragments, which identify the wild dominant or the mutant recessive allele, respectively. The results section shows how the combinations among the *MC1R* and *ASIP* genotypes determined the real genetic coat colors. To confirm the assigned genotypes, thus establishing the reliability of the used methods, 40 samples randomly chosen were sequenced in forward and reverse direction by a commercial service. Once the individual genetic color had been assigned, the genotype of each subject was compared with the color reported on the individual card after visual observation at the time of capture and identification, to verify the correspondence.

#### Statistical Analysis

Allele frequencies were calculated by direct count of the detected genotypes in the *MC1R* and *ASIP* genes.

The observed genotypes and the horse coat color were compared using the χ2 test by means of the R statistical software Version 4.3.2 [22].

## 3. Results

Genotyping results showed the genetic coat color distribution in the examined population. Sequencing of the randomly chosen samples confirmed the assigned genotypes. Thanks to their reduced costs and simple method of execution, PCR/RFLP techniques can be a valid solution to be applied routinely for faster and cheaper analysis compared to sequencing, while ensuring correct coat color assignment.

*MC1R* gene genotyping started with amplification of the 320 bp fragment, corresponding to position 36,979,377–36,979,697 of the latest EquCab3.0 (GCF_002863925.1) horse genome assembly. Digestion by *TaqI* endonuclease detected the C > T transition (rs68458866), which allowed us to distinguish the wildtype dominant C from the mutant recessive T allele. The resultant genotype set was homozygous wild dominant C/C, heterozygous C/T and homozygous mutant recessive T/T. The majority of the analyzed Sarcidano Horses carried the double recessive T/T genotype (58 heads, corresponding to 64% of the total studied population), 27 horses (30%) carried the heterozygous genotype C/T, and only 5 horses carried the homozygous wild dominant genotype C/C, equivalent to 6% of the studied population. The allele frequency was 79% for the T and 21% for the C allele (Table 1).

The causative mutation falls at position chr:3: 36,979,560, producing TCC or TTC as possible codons within the protein chain, at position 83 (NCBI Reference Sequence: NP_001108006.1), resulting in a serine being replaced with a phenylalanine [14,23]. This single mutation in the proteins’ primary structure is expected to alter the alpha helix structure, thus producing a defective functioning of the MC1 receptor, which becomes unable to be activated by the melanocyte-stimulating hormone (MSH). This failure to function leads to pheomelanin in place of eumelanin production within melanocytes. Thereby, a Black horse exhibits at least a wildtype C allele, while a Chestnut one carries only a T/T genotype. Consequently, Agouti locus determines whether a horse carrying at least a C allele will be Bay (if it exhibits at least one recessive type 91 allele) or Black (if it carries a homozygous 91/91 genotype); there is no effect of the *ASIP* gene genotype on chestnut-based horses.

*ASIP* gene genotyping was conducted directly through amplification by PCR of the Exon 3, producing a 102/91-bp fragment. Indeed, a polymorphic 11-bp deletion (rs396813234) allowed us to identify the three available genotype sets based on the resultant fragment size. The entire (102-bp) nucleotide sequence corresponds to the dominant wildtype allele, here named as “102”, while the 11-bp deletion identifies the mutant recessive allele, here named as “91”. Consequently, the corresponding genotype set for the *ASIP* gene was 102/102 for the wildtype homozygous dominant, 102/91 for the heterozygous and 91/91 for the homozygous mutant recessive genotypes.

The *ASIP* gene genotype distribution exhibited only two horses carrying the wildtype dominant 102/102 genotype (corresponding to 2% of the studied population), while the main part of the population exhibited the recessive 91/91 genotype (62 horses, corresponding to 69% of the analyzed Sarcidano Horses) and 26 subjects (29%) resulted heterozygous 102/91. Allele frequency resulted 83% for the recessive 91 allele and 17% for the wildtype 102 allele. Both the loci showed a very low frequency of the wild dominant allele and a very high rate of the mutant recessive type (Table 1).

**Figure 2:**
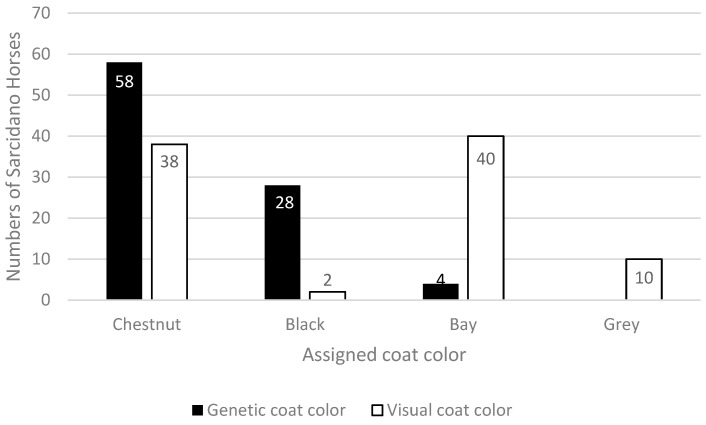
Differences between genetic and visual coat color assignment in the 90 studied Sarcidano Horses. Gray horses are presented only as having a phenotypic coat color as they are carriers of a basic genetic color, which is highlighted by genetic analysis.

From a genetic point of view, more than half of the analyzed population resulted Chestnut, well over a quarter were Blacks and a very small portion were Bays. Inconsistencies in visual coat color registration emerged when these results were matched with the phenotypically recorded data, producing an altered distribution of coat colors in the studied population. Indeed, from a phenotypic point of view, the 90 Sarcidano Horses were registered as 38 Chestnuts, 40 Bays, only 2 Blacks, and 10 Grays. Gray horses were recorded simply as “grey”, without indication of the possible true color they carried (Figure 2).

The error rate (calculated as the number of incorrectly classified color phenotypes in the total number of genetically assigned coat colors) was 53.4%, including the gray horses. This large discrepancy between phenotypic and genetic data highlighted how visual observation can greatly alter coat color assignment, leading to incorrect registration of individual data (Table 2 and Supplementary Table S1).

Different genotype combinations were found for each coat color, as reported in Table 3.

These combinations produced some differences in the color shades, varying from light to dark within the basic color set, that could be responsible for the inconsistencies in visual color recognition. Chestnuts resulted in the following combinations: T/T + 91/91 (59% of the total chestnuts), T/T + 102/91 (38%) and T/T + 102/102 (3%). The most represented genotype combination in the studied Sarcidano Horses resulted T/T + 91/91, producing a liver Chestnut, seven of which were visually identified and recorded as Bay. Of the 22 horses carrying the T/T + 102/91 combination, producing a phenotypic lighter-sorrel Chestnuts, 5 were visually registered as Bays. In Black horses too, different genotype combinations were found: 23 were C/T + 91/91 (82% of the Blacks), 21 of which were visually registered as Dark Bays, while 5 were C/C + 91/91, of which 4 were visually recognized as Bays. This important discrepancy in recognizing the black coat color has different possible explanations. The non-black areas of a Bay horse can range from a light brown to near-black, whereas Black horses can range from a sun-faded brown to jet black. This range of possible shades can make it difficult to pinpoint the exact coat color and create confusion when recording [24,25,26]. While all other mammals, including horses, have the genetic potential to be black, certain breed standards do not recognise this possibility and classify all dark coats as Dark Bay. Consequently, a very dark horse is registered as Dark Bay in some breeds, and many people who fill out individual forms apply this rule to all breeds. However, the genetics of a Bay coat are different to those of a Black one, thus preventing the coat color from being correctly assigned.

In this study, only 4 horses were found to be genetically Bays, while visually, as many as 40 heads were registered with this coat. All four of the Bays carried the heterozygous genotype at both the loci, C/T + 102/91. The other possible allele combinations for a Bay coat color are C/C + 102/102, C/C + 102/91 and C/T + 102/102, but none of them were found in the studied Sarcidano Horses. All these results are summarized in Table 4.

## 4. Discussion

The found inconsistencies in visual vs. genetic coat color recognition means that a subjective perception of shadings can alter the correct identification, making visual evaluation not completely reliable, as the same horse could be identified differently by different evaluators.

The effects of both personal and environmental elements, including age, season, housing type, and diet can make the coat color identification process challenging [9]. As a result, a black horse kept outside all the time may show signs of “fading” in their coat, which may be mistaken for a Dark Brown horse. On the other hand, a Dark Bay horse’s coat may be confused with a black coat that has faded from sunlight because it has a black mane, tail, and legs, and very dark brown or reddish hair around the head, neck, back, and hips [25,26].

Instead, it is crucial to identify coat colors accurately and to correctly report them in the Stud Book, for legal and medical certification, and for the correct prediction of inheritable coat colors, which can only be achieved with genetic investigation of the *MC1R* and *ASIP* loci genotypes [27].

Another source of confusion concerns Gray horses, as they are born Black, Chestnut or Bay but gradually lose color in their coat as they age [28]. This means that an adult Gray horse masks its true color, and it is not clear what color its offspring will inherit. However, since the gray locus is epistatic to the above base coat color genes, if a horse is gray, one can be assured that it has at least one gray parent, but it is impossible to know what color its foals will be without knowing what genetic color they carry [29]. The same can be said also for Chestnuts and Bays. Indeed, Chestnut horses, although they are surely carriers of the T/T genotype at the MC1R locus, do not externally show which genotype they have at the ASIP locus; therefore, they can generate offspring of any coat color base, depending on the partner’s genotype.

Even Bay horses do not display the genotype they carry at the ASIP locus and are consequently able to transmit different variants of ASIP alleles; a homozygous 102/102 horse will necessarily pass on the 102 allele to its offspring, while a heterozygous horse (102/91) will have a 50% chance of passing on the 102 allele.

Therefore, when the individual coat color is genetically identified, it is possible to know without any doubt, and from birth, what type of foal each horse will be able to generate, in the absence of other color modifiers, as is the case for the Sarcidano Horse. From homozygous MC1R and ASIP loci parents (C/C + 102/102), all offspring will be Bay. From C/C + 102/91 parents, all offspring will be dark based, but could be both Dark Bay and true Black. From C/T + 102/102 parents, the offspring will be Bay as they receive homozygous wild type ASIP alleles, diluting black body color, so that they can be visually appear more red or more black depending on coat color tone and different external and individual factors, such as age, environmental conditions, and nutritional status [30]. Finally, from heterozygous C/T + 102/91 parents, any color offspring are possible.

## 5. Conclusions

To conclude, this study tested the genetic coat colors of the Sarcidano Horse and revealed a high percentage of discrepancies that were assigned phenotypically. Furthermore, this research contributes to laying out a reliable, easy, and low-cost method for the identification of coat color in horses, avoiding incorrect recording of collected data and uncertain heritable color prediction. Given the wild nature of the Sarcidano Horse, visual observation of coat color is not always an easy activity, since it occurs outdoors, under any weather condition, without guaranteeing optimal observation time, due to the capture requirements. Inconsistencies in coat color registration are a common problem for many horse breeds, in which assignment only through observation can lead to inaccurate attribution of lineage. Incorrect parentage may be a consequence of the observed discrepancies, which could have a detrimental effect on the health and variability of such a small population, but the main goal should be to preserve all of these ancient native breeds. The introduction of routine paternity testing would be useful to avoid incorrect attribution of lineage, but it still entails high costs and would not solve the problem of incorrect color attributions. Enhanced training of technicians for visual coat color identification is surely recommended, but it should be highlighted that discrepancies in coat color assignment could be completely remedied if genetic testing was routinely carried out. Although further initiatives to defend this important genetic resource would be desirable, at present, only a few breeders and local authorities and passionate researchers are actively involved in increasing interest in this small population.

## Figures and Tables

**Table 1:** Genotype and allele distribution of the *MC1R* and *ASIP* genes polymorphisms according to the genetic coat color assignment of the 90 genotyped Sarcidano Horses.

		*MC1R*					*ASIP*				
		Genotypes			Alleles		Genotypes			Alleles	
		C/C	C/T	T/T	C	T	102/102	102/91	91/91	102	91
Coat color	Black	5	23	0	33	23	0	0	28	0	56
	Bay	0	4	0	4	4	0	4	0	4	4
	Chestnut	0	0	58	0	116	2	22	34	26	90

**Table 2:** Difference between genotypes and visual observation in the coat color of the 90 Sarcidano Horses.

Genotypes		Visual Observation	
MC1R	ASIP	Correct (%)	Incorrect (%)
C/C	91/91	60.0	40.0
T/C	91/102	75.0	25.0
T/C	91/91	0.0	100.0
T/T	91/91	61.8	38.2
T/T	91/102	59.1	40.9
T/T	102/102	100.0	0.0
Total		46.6	53.4

**Table 3:** Base coat color phenotypes and the combined distribution of *MC1R* and *ASIP* loci genotypes in the 90 genotyped Sarcidano Horses.

		*MC1R* Genotype		
		C/C	C/T	T/T
*ASIP* genotype	102/102	0 Bay	0 Bay	2 Chestnut
	102/91	0 Bay	4 Bay	22 Chestnut *
	91/91	5 Black	23 Black **	34 Chestnut ***

* Three of which were phenotypically gray; ** One of which was phenotypically gray; *** Six of which were phenotypically gray.

**Table 4:** Cumulative combined distribution of visual and genetic coat color recorded in the 90 studied Sarcidano Horses expressed as a percentage.

		Visual Coat Color (VCC)				
		Bay	Gray	Black	Chestnut	Total GCC
Genetic Coat Color (GCC)	Bay	0.03	0.00	0.00	0.01	0.04
	Black	0.28	0.00	0.02	0.01	0.31
	Chestnut	0.14	0.11	0.00	0.40	0.65
Total VCC		0.45	0.11	0.02	0.42	

## Data Availability

The data presented in this study are available upon request from the corresponding author.

## Supplementary Materials

The following supporting information can be downloaded at: https://www.mdpi.com/article/10.3390/ani14040543/s1, Table S1: All individual coat colors identified by genotyping and visually assigned.
