# Supplementary material for: Discrepancies between Genetic and Visual Coat Color Assignment in Sarcidano Horse
Source: Animals (Basel). 2024 Feb 6;14(4):543. doi: 10.3390/ani14040543 (PMC10885979; doi:10.3390/ani14040543)
Supplement: Supplementary file 1 [file animals-14-00543-s001.zip › animals-2760499-supplementary.pdf]

Table S1. All individual coat colors identified by genotyping and visually assigned.

ID	ASIP	MC1R	genetic color	phenotipic color assigned
S1	91/91	T/T	Chestnut	Bay
S2	91/102	T/T	Chestnut	Bay
S3	91/102	T/T	Chestnut	Chestnut
S4	91/102	T/T	Chestnut	Grey
S5	91/91	T/T	Chestnut	Bay
S6	91/91	T/T	Chestnut	Chestnut
S10	91/91	T/T	Chestnut	Bay
S11	91/102	T/T	Chestnut	Chestnut
S12	91/91	T/T	Chestnut	Chestnut
S13	91/91	T/T	Chestnut	Grey
S14	91/91	T/T	Chestnut	Chestnut
S15	91/91	T/C	Black	Bay
S16	91/91	T/T	Chestnut	Grey
S17	91/91	C/C	Black	Black
S18	91/91	T/C	Black	Chestnut
S19	91/91	T/C	Black	Bay
S20	91/91	T/T	Chestnut	Chestnut
S21	91/91	T/T	Chestnut	Chestnut
S22	91/91	T/T	Chestnut	Chestnut
S23	91/91	T/T	Chestnut	Chestnut
S24	91/91	T/C	Black	Bay
S25	91/91	T/C	Black	Bay
S26	91/91	C/C	Black	Bay
S27	91/102	T/T	Chestnut	Bay
S28	91/91	T/T	Chestnut	Chestnut
S29	91/91	C/C	Black	Bay
S30	91/91	C/C	Black	Black
S31	91/102	T/T	Chestnut	Chestnut
S32	91/102	T/C	Bay	Bay
S33	91/91	T/C	Black	Bay
S34	91/102	T/T	Chestnut	Grey
S35	91/91	T/T	Chestnut	Chestnut
S36	91/91	T/C	Black	Bay
S37	91/91	T/T	Chestnut	Chestnut
S38	91/102	T/T	Chestnut	Chestnut
S39	91/91	T/C	Black	Bay
S40	91/102	T/C	Bay	Chestnut
S41	91/91	C/C	Black	Bay
S42	102/102	T/T	Chestnut	Chestnut

S43	91/91	T/T	Chestnut	Grey
S44	91/102	T/T	Chestnut	Chestnut
S45	91/91	T/T	Chestnut	Chestnut
S46	91/102	T/T	Chestnut	Bay
S47	91/91	T/T	Chestnut	Chestnut
S48	91/102	T/T	Chestnut	Chestnut
S49	91/91	T/T	Chestnut	Chestnut
S50	91/91	T/T	Chestnut	Grey
S51	91/91	T/T	Chestnut	Grey
S53	91/91	T/C	Black	Bay
S54	91/102	T/T	Chestnut	Grey
S55	91/91	T/T	Chestnut	Bay
S56	91/102	T/T	Chestnut	Chestnut
S57	91/91	T/T	Chestnut	Chestnut
S58	91/91	T/C	Black	Bay
S59	91/102	T/C	Bay	Bay
S60	91/102	T/T	Chestnut	Chestnut
S61	91/102	T/T	Chestnut	Grey
S62	91/91	T/C	Black	Bay
S63	91/91	T/C	Black	Bay
S64	91/102	T/T	Chestnut	Chestnut
S65	91/102	T/T	Chestnut	Bay
S66	91/102	T/C	Bay	Bay
S67	91/91	T/T	Chestnut	Bay
S68	91/91	T/C	Black	Bay
S69	91/91	T/C	Black	Bay
S70	91/102	T/T	Chestnut	Bay
S71	91/91	T/C	Black	Bay
S72	91/91	T/C	Black	Bay
S73	91/91	T/T	Chestnut	Chestnut
S74	91/102	T/T	Chestnut	Chestnut
S75	91/91	T/T	Chestnut	Chestnut
S76	91/91	T/C	Black	Bay
S77	91/91	T/C	Black	Bay
S79	91/91	T/C	Black	Bay
S80	91/91	T/T	Chestnut	Bay
S81	91/91	T/T	Chestnut	Chestnut
S82	91/102	T/T	Chestnut	Chestnut
S83	91/91	T/T	Chestnut	Chestnut
S84	91/91	T/C	Black	Bay
S86	91/102	T/T	Chestnut	Chestnut
S87	91/102	T/T	Chestnut	Chestnut
S88	91/91	T/T	Chestnut	Grey

S89	102/102	T/T	Chestnut	Chestnut
S90	91/91	T/T	Chestnut	Chestnut
S91	91/91	T/T	Chestnut	Bay
S92	91/91	T/T	Chestnut	Chestnut
S93	91/91	T/T	Chestnut	Chestnut
S94	91/91	T/C	Black	Bay
S95	91/91	T/C	Black	Bay
S97	91/91	T/C	Black	Bay
